# Is Minimally Invasive Surgery the Standard of Care for Ventral Hernia Repair?

**DOI:** 10.5041/RMMJ.10540

**Published:** 2025-01-30

**Authors:** Kaushik Bhattacharya

**Affiliations:** Associate Professor, Department of Surgery, Mata Gujri Memorial Medical College and LSK Hospital, Kishanganj, Bihar, India

**Keywords:** Minimally invasive surgery, open intraperitoneal onlay mesh, ventral hernia

When patients undergoing ventral or incisional hernia repair are reoperated for recurrence with an incidence rate of 16.0% following open repair and 18.8% following minimally invasive repair,^1^ it is time for re-evaluation of the real benefit of laparoscopy in ventral hernia repair. Approximately 1 in 10 patients experienced decision regret following ventral and incisional hernia repair with laparoscopy, as the much-hyped and anticipated benefits of faster recovery and minimal pain did not match their actual postoperative experiences.^2^ Open intraperitoneal onlay mesh (IPOM) repair for small ventral hernia has been found to provide better results than laparoscopic IPOM. This is attributed to the shorter operative time, no port hernia risk, and the ability to perform excess skin excision while achieving better umbilical reconstruction, all with only a single incision.^3^

A significant issue regarding laparoscopic ventral hernia repair (LVHR) is the potential for postoperative seroma formation, which occurs in nearly all patients in the area anterior to the mesh during the early postoperative period. This can lead to pain, poor aesthetic outcomes, discomfort, surgical site infections, and an increased length of hospitalization.^4^ The risk of pseudo hernia formation and postoperative hernia bulge is a challenge for LVHR, apart from the risk of inadvertent enterotomy during LVHR, ranging between 1% and 11%.^5^

Unlike decision regret after LVHR, most patients in a recent study reported highly satisfactory outcomes and quality of life after open mesh repair of a midline incisional hernia when assessed with Patients-Reported Outcome Measures (PROM).^6^ Almost 70% of the surgical literature showing the advantages and benefits of LVHR over open surgery have conflicts of interest^7^,^8^; hence, the real picture is never brought out. The various hernia societies worldwide have their vested interests in promoting LVHR.^9^ Furthermore, the mesh manufacturing industry has significantly influenced hernia repair guidelines in several ways, primarily through research funding, marketing efforts, and surgeon education. The growth of the hernia mesh industry has been primarily due to mesh manufacturers and their funding of clinical trials, formulation of HerniaSurge guidelines,^10^ and influence in studies to promote the use of their products.^8^,^11^

In the end, the issue that every herniologist must face relates to the ethics and rationality of routinely preferring one surgical procedure over another without questioning the added value and possible adverse events.

## Abbreviations

IPOM: open intraperitoneal onlay mesh
LVHR: laparoscopic ventral hernia repair
